# Increase in Reported Crimean-Congo Hemorrhagic Fever Cases — Country of Georgia, 2014

**Published:** 2015-03-06

**Authors:** Ashley L. Greiner, Nana Mamuchishvili, Stephanie J. Salyer, Kendra Stauffer, Marika Geleishvili, Khatuna Zakhashvili, Juliette Morgan

**Affiliations:** 1 Epidemic Intelligence Service, CDC; 2 Division of Global Health Protection, Center for Global Health, CDC; 3 National Centers for Disease Control and Public Health, Country of Georgia; 4 South Caucasus Country Office, Center for Global Health, CDC

During January–September 2014, Georgia’s National Centers for Disease Control and Public Health (NCDC) detected 22 cases of Crimean-Congo hemorrhagic fever (CCHF) in the country. CCHF is caused by infection with a tickborne virus of the *Bunyaviridae* family (1–3). Transmission occurs from the bite of an infected tick or from crushing an infected tick with bare skin. Secondary transmission can result from contact with blood or tissues of infected animals and humans. CCHF initially manifests as a nonspecific febrile illness that progresses to a hemorrhagic phase, marked by rapidly developing symptoms leading to multiorgan failure, shock, and death in severe cases (2). The clinical severity, transmissibility, and infectiousness of CCHF are responsible for its categorization as a viral hemorrhagic fever high-priority bioterrorism agent (4).

The first case of CCHF in Georgia was detected in 2009 when Georgia initiated passive CCHF surveillance. During 2009–2013, the surveillance system detected a median of one case per year (range = 0–13 cases). A case is defined as fever (temperature >100.4^o^F [>38^o^C]), at least one hemorrhagic sign (petechial or purpural rash, bleeding, or thrombocytopenia), and a positive CCHF nucleic acid amplification test or anti-CCHF immunoglobulin M titer in a resident of Georgia. Although CCHF is endemic in the Caucasus region, the 22 cases detected in the first 9 months of 2014 are the highest number of cases reported in that time frame, suggesting a change in either the epidemiology of the disease or the national surveillance system.

To determine the source, mode of transmission, and risk factors for each case, NCDC in collaboration with CDC examined 2014 surveillance data. Case reports were extracted from NCDC’s Electronic Integrated Disease Surveillance System. Additionally, NCDC and national reference laboratory staff members were interviewed to identify changes in disease surveillance that might have increased the system’s sensitivity.

Among 22 patients, the mean age was 45 years (range = 4–77 years); 13 (59%) were male. Most (91%) cases occurred during May 1–August 31; 18 (82%) occurred in rural villages. Preceding their illness, 14 (64%) patients reported a tick bite or removal, and three (14%) reported exposure to animal blood. The mean incubation period was 4 days (range = 1–17 days). Of those responding, 19 of 21 (90%) patients had fever, 17 of 18 (94%) had thrombocytopenia, and 13 of 20 (65%) had bleeding. The case-fatality rate was 14%. Interviews revealed recent activities that have led to increased CCHF testing; these have included a nationwide educational campaign in 2012 to increase CCHF physician awareness and testing for CCHF in two acute febrile illness studies through NCDC and other partners during 2008–2011, and from 2014 to present (5).

Since surveillance for CCHF began in Georgia in 2009, annual case counts have increased progressively. This trend might reflect improving surveillance sensitivity, which could have been stimulated by the educational campaign and acute febrile illness studies. Thus, the 2014 increase in cases might be an artifact of improved surveillance system sensitivity, rather than an actual increase in incidence. Overall, the increasing annual case count highlights the importance of ongoing CCHF surveillance in Georgia as well as expanding current efforts to continue improving surveillance sensitivity.

Despite increased surveillance system sensitivity, underreporting likely still exists. Hemorrhagic signs in CCHF are a predictor of mortality (2,6). In 2014, CCHF patients in Georgia had a higher frequency of hemorrhagic signs compared with those displayed by CCHF patients in neighboring Turkey during 2002–2007 (65% versus 23%, respectively) (7). This might indicate that a more virulent strain of the virus exists in Georgia, or the greater severity of the reported cases could indicate that milder CCHF cases are not being detected. To reduce the likelihood of underreporting, ongoing physician educational campaigns should encourage CCHF diagnostic testing in patients with milder symptoms.

Human exposure to infected ticks and animals are likely principal risk factors for CCHF transmission in Georgia. The seasonal distribution of CCHF cases in Georgia corresponds to months of predicted peak tick activity. Additionally, all 2014 CCHF patients resided along a major herding corridor in Georgia. Public health interventions in Georgia need to target these exposures. Specifically, ongoing educational campaigns might intensify focus on 1) preventing tick exposure and encouraging safe tick handling practices among herders, farmers, and veterinarians; and 2) minimizing contact with infected animal blood and tissues among herders, slaughterhouse workers, veterinarians, and health care workers.

A seroprevalence survey in the rural villages reporting a 2014 CCHF case is under way. Further investigations in Georgia should be considered to determine whether CCHF incidence exceeds that reported through the surveillance system and to estimate the overall burden of CCHF in Georgia. Additionally, cattle and tick testing in the affected villages should be considered. These findings will help direct future public health planning with the goal of reducing CCHF infection in the Georgia population.
